# Metabolomics Approach for the Analysis of Resistance of Four Tomato Genotypes (*Solanum Lycopersicum* L.) to Root-Knot Nematodes (*Meloidogyne Incognita*)

**DOI:** 10.1515/biol-2019-0016

**Published:** 2019-04-06

**Authors:** Enik Nurlaili Afifah, Rudi Hari Murti, Tri Rini Nuringtyas

**Affiliations:** 1 Faculty of Agriculture, Universitas Gadjah Mada, Yogyakarta Indonesia; 2 Faculty of Biology, Universitas Gadjah Mada, Yogyakarta Indonesia

**Keywords:** NMR, metabolomics, defense mechanism, Solanum lycopersicum, nematode

## Abstract

Metabolomics allows the identification of biochemical markers that have important roles in plant resistance to pests and diseases by which breeders can select plants based on differences in these compounds. This study examines the range of compounds associated with plant defense against nematodes. Resistant tomato genotypes, GM2 and F1 (GM2 × Hawai 7996), and susceptible genotypes, Gondol Putih and Gondol Hijau, were used in this study. Peroxidase activity was measured colorimetrically using a spectrophotometer. ^1^H-NMR (nuclear magnetic resonance) spectroscopy combined with orthogonal projections to latent structures discriminant analysis was used to analyze the metabolites involved in the tomato-nematode interactions. Identified signals were semi-quantitatively calculated by scaling the intensity of the ^1^H-NMR to the signals of an internal standard (trimethyl silyl-3-propionic acid) at 0.00 ppm. Resistant plants showed a higher peroxidase activity than susceptible plants. Chemical compounds that differentiated between susceptible and resistant plants were glucose and caffeic acid. Resistant tomatoes were observed to have seven times higher level of glucose than susceptible plants. Glucose is the primary metabolite that acts in the signaling pathways in plant defense mechanisms. Caffeic acid is one of the phenolic compounds alleged to have a negative effect on the nematode.

## 1 Background

Tomato is a vegetable crop that is often cultivated by farmers around the world because of its health benefits due to its high content of vitamin C, carotenoids, folate, and phytochemicals that help to prevent cancer and other diseases [1]. However, recent data have indicated a decrease in tomato productivity in Indonesia due to diseases and pests [2] including root-knot nematodes. Root-knot nematodes (*Meloidogyne* spp.) cause root damage by developing galls. Galls are formed due to hypertrophy and hyperplasia within the roots, thereby disturbing vessel transport and inhibiting water and nutrient translocation, which leads to chlorosis. The chlorosis caused by the nematodes affects photosynthesis, thereby reducing the tomato yield [3].

Nematodes are generally controlled using chemicals such as nematicides; however, this has been proven to have a negative effect on the environment and leave residues in foods. The use of resistant plants has been considered as a more effective strategy to control root-knot nematodes [4] because of low cost and risk. Thus, plant breeders aim at improving potential, harvestable and marketable yield by selecting plants resistant to diseases and pests [5]. To achieve this goal, breeders must be aware of mechanisms of resistance and the types of genetic resistance and then produce new resistant cultivars [6].

A metabolomics approach can be applied to support breeding programs. This approach allows plant breeders to identify compounds that play an important role in plant resistance to pests and diseases. Resistant plants produce several biologically active chemicals and secondary metabolites that enable them to be more resistant to pests and diseases. López-Gresa et al. compared the metabolic profiles of inoculated plants with appropriate controls using nuclear magnetic resonance (NMR) spectroscopy to unravel the biochemical pathways associated with resistance of tomato plants to citrus exocortis viroid [7]. Roman et al. also used NMR spectroscopy to detect compounds associated with the resistance of tomato plants to thrips [8]. However, there are only a few studies that have applied metabolomics to observe the interactions between tomato plants which are either susceptible or resistant to infections and root-knot nematodes. An earlier study by Eloh et al. investigated metabolomic profiles of nematode interactions with tomato plants using gas chromatography mass spectrometry to identify the different compounds before and after inoculation [9]. However, the study did not determine the metabolite differences between resistant and susceptible plants.

One of the resistance mechanisms to root-knot nematodes involves peroxidase enzymes. Medeiros et al. described that peroxidases contribute to the resistance level of tomato plant to root-knot nematodes by producing toxins, inhibiting nematode development and penetration into the roots [10]. Therefore, in addition to metabolomic analysis, it is also necessary to evaluate the peroxidase activity to determine the presence of these compounds in resistant plants attacked by nematodes.

Murti et al. studied genotypes resistant to nematodes, and found that the GM2 accession was the most resistant to nematodes based on the lower root gall intensity, juvenile 2 (J2) number in the root and soil, and egg mass number. They also studied genetic inheritance of the resistance to nematodes [11]. The nematode resistance in GM2 is controlled by two genes. Therefore, the GM2 tomato genotype is resistant to nematodes, whereas both Gondol Putih and Gondol Hijau are susceptible. This study also used resistant genotypes (F1) that resulted from the crossing between GM2 and Hawaii 7996. In the present study, these genotypes, in combination with metabolomics, were used to identify chemical compounds that are involved in the chemical resistance mechanism to root-knot nematodes.

## 2 Materials and Methods

### 2.1 Nematode Inoculum

Nematodes collected from infected tomato roots cultivated in Kaliurang, Sleman Yogyakarta, Indonesia that were infected with *Meloidogyne incognita* were used in this study. The nematodes that were required in this study were 2000 J2 per plant. An inoculum was produced by cutting the roots into 20 to 30 mm pieces that were then placed in 200 ml of 0.5% -1% NaOCl and shaken for 4 min. Subsequently, the suspension was filtered through 400 and 500 mesh to collect eggs that were then washed in sterile water to remove NaOCl. The collected eggs were placed in a water suspension for 3-4 days until they developed into J2 juveniles. This suspension was then used as an inoculum [12]. The suspension was adjusted to 500 J2/ml to apply 2000 J2 per plant.

### 2.2 Plant Material and Experimental Procedure

The experiment was carried out in a greenhouse of the Faculty of Agriculture, Universitas Gadjah Mada (UGM). Tomato (*Solanum lycopersium* L.) plants of the nematode-resistant tomato varieties, GM2 and F1 (resulting from GM2 × Hawai 7996), and the susceptible varieties, Gondol Putih and Gondol Hijau, were used. Seeds were sown in sterile soil medium enriched with fertilizer (NPK 1:1:1) at a ratio 1:50 (w/w). The seedlings (21 days after sowing) were individually transplanted into polybags (130×130 mm). The soil medium was also enriched with fertilizer (NPK 1:1:1) at ratio 1:50 (w/w) and compost with ratio 3:1 (w/w). Two treatments, a control (application of only water) and root-knot nematode applications (2000 J2 juveniles per plant), were applied to all genotypes 7 days after transplantation. There were five replications per genotype, and the plants were harvested 45 days after the transplantation. At harvest, the roots were cleaned of growth medium using distilled water and examined and scored for root gall intensity [based on Zec et al.’s (1971) method [13]], root volume (measured with gravimetric approach), root length, and root and shoot weight. An amount of 5 g of fresh roots per plant was collected for NMR and peroxide analysis. For NMR analysis, liquid nitrogen was added to the root samples and immediately ground in a pre-cooled mortar. The samples were freeze-dried for 2 days to remove the water content.

### 2.3 Peroxidase Analysis

Assessing peroxidase activity used five replicates per plant. Fresh root tissue (1 g) was crushed using a mortar and then 0.01 M phosphate buffer (pH 6) was added at a ratio of 1:4 (w/v). The mixture was then centrifuged at 5000 ppm for 30 min at 4°C, and then filtered through a Whatman filter paper. The enzyme activity was observed by setting up two tubes; the first tube contained 5 ml of root extract and 5 ml of pyrogallol, and the second tube consisted of 5 ml of root extract, 5 ml of 0.5 M pyrogallol and 1% H_2_O_2_. Each tube was mixed for 5-10 s. The absorbance of each tube was measured at 420 nm using a spectrophotometer until the value become constant [14].

### 2.4 Metabolomic Analysis by 1H-NMR

The metabolomic analysis of root extract in this experiment was based on the modified method of López-Gresa et al. [7]. This analysis used five replicates per plant. Approximately 50 mg of freeze-dried tomato root sample was placed in a 2 ml Eppendorf tube, and then mixed with 1 ml of methanol-d4 containing 0.05% trimethyl silyl-3-propionic acid (TMSP). The samples were vortexed for 20 min, sonicated for 20 min and then centrifuged at 13.000 rpm for 10 min. After centrifugation, ~800 ml of the suspension was taken for NMR analysis. The NMR condition was set based on a previous study [15]. ^1^H-NMR spectra were recorded at 30˚C with a 500 MHz Jeol NMR spectrometer (Jeol, USA Inc.). Each ^1^H NMR spectrum consisted of 26 s acquisition time and 128 scans requiring 10 min, with the following parameters: 0.16 Hz/point, pulse width of 30 (11.3 ms), and 1.5s relaxation delay. A pre-saturation sequence was used to suppress residual water signal with low power selective irradiation at the water frequency during the recycle delay. Free Induction decays were Fourier transformed with a 0.3 Hz line broadening. The resulting spectra were manually phased, baseline corrected, and calibrated to TMSP at 0.0 ppm using the MNOVA software.

### 2.5 Data Analysis

The ^1^H-NMR signals were identified using the MNOVA software. Spectral intensities were scaled to total intensity and reduced to the integrated region of equal width (0.04 ppm) from δ 0.4 to 10.0. The residual of water (the regions of δ 4.7- 4.9) and methanol (the regions of δ 3.28 – 3.34) were excluded from the analysis. Multivariate data analysis was conducted using principal component analysis (PCA) followed by orthogonal projections to latent structures discriminant analysis (OPLS-DA) using the soft independent modeling of class analogy (SIMCA-P) software (version 11.0, Umetrics, Umeå, Sweden). Scaling was set to pareto. Validation of OPLS-DA was done by permutation analysis and CV-ANOVA.

ANOVA was performed using the R software to determine the differences between tomato genotypes in terms of metabolite concentrations, root gall intensity, root gall number, and shoot and root weights. All the data were transformed to before analysis, and further analyzed using Tukey’s HSD tests to determine significance differences between mean value at P = 0.05.

## 3 Results

### 3.1 Root Gall Intensity and Tomato Morphology

**Figure 1:**
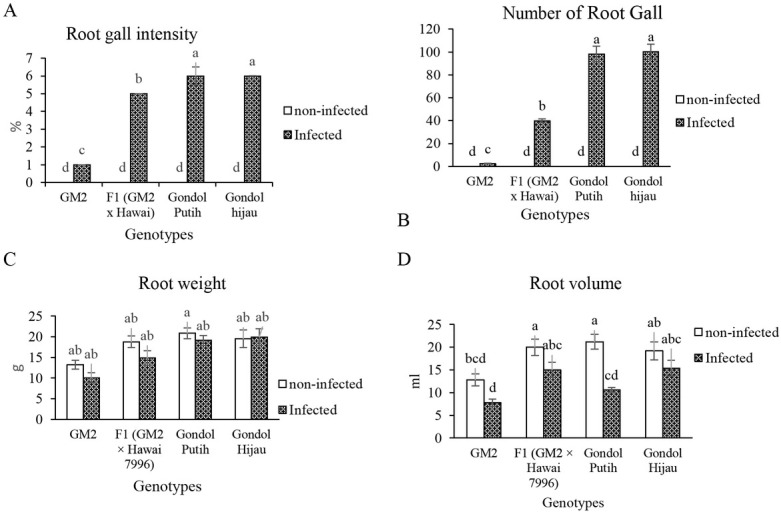
(A) Root gall intensity, (B) root gall number, (C) Root weight, and (D) Root volume of nematode-resistant and susceptible tomato cultivars. Bars with the same letter are not significantly different at 5% level according to Tukey’s HSD test.

Regarding root gall intensities and root gall number, significant differences were observed between resistant genotypes (GM2 and F1) and susceptible genotypes (Gondol Putih and Gondol Hijau; Fig. 1). These parameters also showed a significant difference between the resistant genotypes GM2 and F1. GM2 was the more resistant genotype based on low root gall intensity and root gall number compared to F1, whereas F1 was more resistant than Gondol Putih and Gondol Hijau, because F1 had lower root gall intensity and root gall number than the susceptible genotypes.

Regarding root weight and volume, there were no significant differences between resistant plants (GM2 and F1) and susceptible genotypes (Gondol Putih and Gondol Hijau) both in the infected and the non-infected treatment. Regarding root volume (infected treatment), statistically significant differences were observed only between GM2 and Gondol Hijau. The resistant genotype GM2 had the lowest root weight and volume due to the small number of root galls. The susceptible plants had larger root weight and volume than the resistant plants, which was due to the higher root gall number.

### 3.2 Peroxidase Activity

**Fig. 2:**
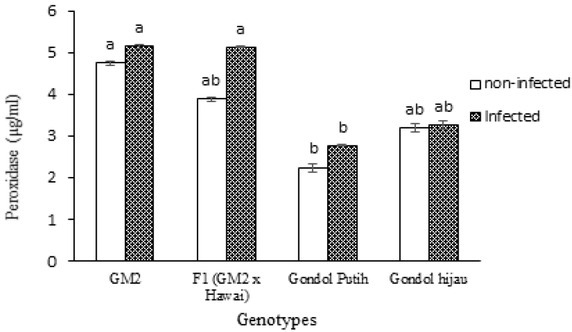
Concentration of peroxidase activity in nematode-resistant and -susceptible tomato genotypes. Bars with the same letter are not significantly different at 5% level according to Tukey’s HSD test.

As shown by the histogram of the peroxidase activity (Fig. 2), plants infected with the nematode exhibited higher peroxide activity than the non-infected, but there was no statistically significant difference between the infected and the non-infected treatments. Figure 2 also shows that there was a significant difference between the resistant genotypes (GM2 and F1) and susceptible genotype (Gondol Putih) in the infected treatment. GM2 and F1 had higher peroxidase activity than Gondol Putih, whereas no statistical difference was observed between resistant genotypes and susceptible genotypes. F1 also showed

no statistical difference when compared with GM2. This indicates that the highest level of peroxidase activity was 4 observed in GM2, followed by F1, and the lowest activity was observed in the susceptible genotypes.

### 3.3 Identification of Metabolites

**Fig. 3:**
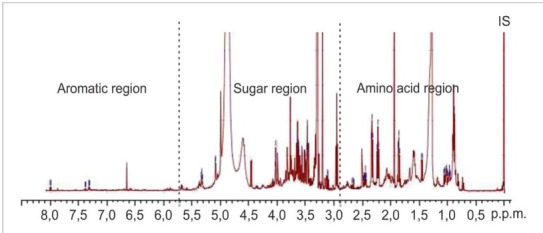
NMR spectra of tomato root extract (genotype: GM2)

Figure 3 shows the ^1^H-NMR spectra of the root samples. The identification of metabolites was based on the NMR spectra of known compounds acquired in previous studies on tomato plants’ NMR database [7, 16, 8]. The spectra could be divided into the following three regions: the amino acid region, the sugar region, and aromatic region, including acetate and succinate. This study resulted in 16 metabolites (Table 1). The amino acid groups included leucine [located in the region δ 0.94 (d, J = 0.7 Hz)], valine [located in the region δ 1.00 (d, *J =* 7.0 Hz); 1.05 (d, *J =* 7.0 Hz)], alanine [located in the region δ 1.45 (d, *J =* 7.2 Hz)], and glycine [in the region δ 3.5 (s)]. The organic acids included acetic acid [in the region δ 1,95 (s)] and γ-aminobutyric acid (GABA) [located in the region 1.88 (m); 2.34 (t, *J =* 7.2 Hz); 2.23 ; 2.96 (t, *J =* 7.08 Hz)], and the sugar group included β-glucose [in the region δ 4.46 (d, *J =* 7.8 Hz)] and α-glucose [located in the region δ 5.09 (d, *J =* 3.76 Hz)].

**Table 1:** Peak assignments for the ^1^H-NMR spectrum of tomato root extracts in MEOD4.

Metabolite	Chemical shift (ppm) and coupling constant (Hz)
Leucine	0.94 (d, *J =* 0.7 Hz)
Valine	1.00 (d, *J =* 7.0 Hz); 1.05 (d, *J =* 7.0 Hz)
Alanine	1.45 (d, *J =* 7.2 Hz)
Acetic acid	1.95 (s)
Succinate	2.52 (s)
GABA (γ-amino-butyric acid)	1.88 (m); 2.34 (t, *J =* 7.2 Hz); 2.23; 2.96 (t, *J =* 7.08 Hz)
Ethanolamine	3.11 (t, *J =* 5.5 Hz)
Choline	3.2 (s)
Glycine	3.5 (s)
β-glucose	4.46 (d, *J =* 7.8 Hz)
α-glucose	5.09 (d, *J =* 3.76 Hz)
Caffeic acid	6.35 (d, *J =* 16.0 Hz); 6.8 (d, *J =* 8.2 Hz); 7.05 (dd, *J =* 8.2, 2.0 Hz); 7.18 (d, *J =* 2.0 Hz); 7.59 (d, *J =* 15.9 Hz)
Fumaric acid	6.66 (s)
PAL (Phenylalanine)	7.31 (d, *J =* 8.0 Hz); 7.39 (d, *J =* 8.0 Hz)
UDPG (Uridine diphospoglucose)	8.00 (d, *J =* 8.0 Hz)
Formic acid	8.5 (s)

*Abbreviations: *J*, the coupling constant; d, doublet; dd, doublet of doublets; m, complex multiplet; s, singlet; t, triplet.

**Fig. 4:**
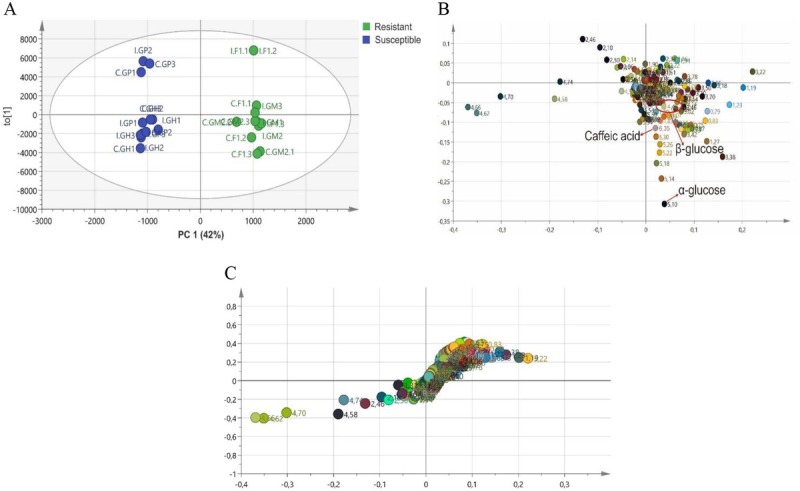
Score (A), loading plot (B) and S-Plot (C) obtained from OPLS-DA of non-infected tomato roots (names starting with “C”) and nematode-infected roots (names starting with “I”). The different colors and values in the loading plot describe the chemical shift (ppm) resulting from the bucket of ^1^H-NMR data.

First, the metabolite profiles based on the ^1^H-NMR spectra of the different treatments (non-infected and infected) and genotypes (GM2, F1, Gondol Putih and Gondol Hijau) were compared using PCA. This analysis did not result in a clear separation between the resistant and susceptible genotypes. This model explained only 22% variation of the data and had a variance R^2^ = 0.672 and a predictive ability Q^2^ = 0.488. Leiss et al. described that Q^2^ is a good value if it is > 0.5 [17]. Therefore, we proceeded to OPLS-DA as shown in Fig. 4A. The OPLS-DA score plot explained 42% of the variation in tomato root metabolites. The score plot revealed the following two groups of plants that were clearly separated from each other: (a) non-infected and infected plants that were resistant to root-knot nematodes located in the positive quadrant of PC1 and (b) non-infected and infected plants that were susceptible and located in the negative quadrant. This model had a variance R^2^ = 0.989 and a predictive ability Q^2^ = 0.549. This validation model used CV-ANOVA and showed significant results (F = 0.43, df = 23, P = 0.02) that indicating a good model. Fig. 4C shows the S-plot in this model; it has high enough reliability as shown by its position far out on the wings of the S.

The difference in the compounds between the susceptible and resistant genotypes was investigated using an OPLS-DA loading plot (Fig. 4B). It was observed that the separation between the susceptible and resistant plants was due to a high concentration of primary metabolites including sugar compounds, secondary metabolites including caffeic acid and other unidentified metabolites. α- and β-glucose and caffeic acid were located in the positive quadrant which was the same as the location of resistant varieties in the score plot.

**Figure 5:**
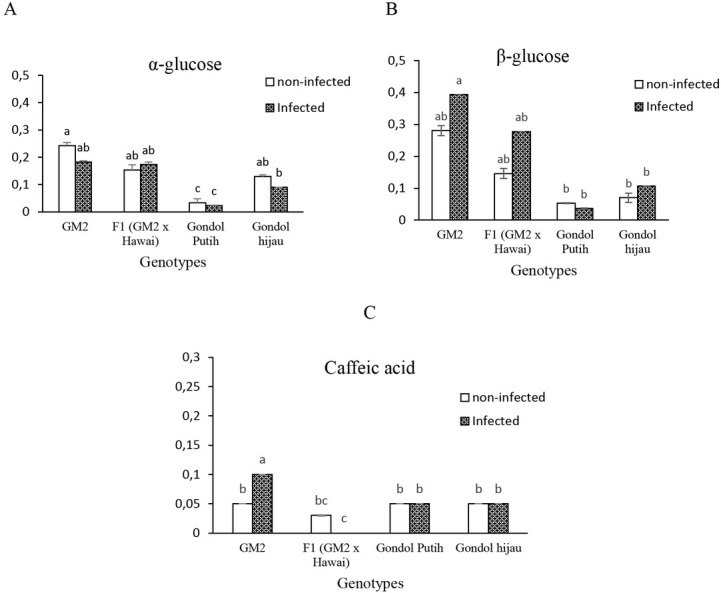
Concentration of α- glucose (A), β-glucose (B), and caffeic acid (C) for the resistant and susceptible tomato plants. Bars with the same letter are not significantly different at 5% level according to Tukey’s HSD test.

Based on the result of semi-quantitative analysis of metabolites, which is an important factor for the separation between the resistant and susceptible plants (Fig. 5), there was no significant difference in both infected and non-infected treatments for the concentration of α-and β-glucose and caffeic acid. Resistant genotypes (GM2

and F1) showed a significantly different concentration of α-glucose compared to that of one susceptible genotype (Gondol Putih), but there were no significant differences with the other susceptible genotype (Gondol Hijau). In the infected treatment, a significant difference was also found in β-glucose concentration between the resistant genotype GM2 and the susceptible genotypes. Caffeic acid was one of the compounds that also affected the separation between the resistant and susceptible plant metabolites. Based on the data (Fig. 5C), caffeic acid concentration was not significantly different between the genotypes in the non-infected treatment, but it was significantly different in the infected treatment. GM2 had a higher concentration of caffeic acid in the nematode infected treatment, and it was statistically significantly different compared to that of F1, Gondol Putih and Gondol Hijau.

## 4 Discussion

GM2 was the most resistant genotype, based on its lowest root gall intensities and root gall number, followed by F1, which exhibited a medium level of resistance to the root-knot nematode based on its lower root gall intensities and root gall number than Gondol Putih and Gondol Hijau (used as susceptible check genotypes). Root galling is one of the characteristics of infestation by *M*. *incognita*, and galls are formed due to hypertrophy and hyperplasia within the roots that disturb vessel transport and inhibit water and nutrient translocation [3]. Giant cells two to three times larger than normal cells from which the nematodes obtain nutrients are formed within the roots. A reduction in root gall intensity indicates that the plants have a defense mechanism to avoid nematode development.

Based on the result of root gall intensity, the most resistant genotype was GM2. Murti et al. described that GM2 was the one genotype that was resistant to the root-knot nematode and has lower root gall intensities [11]. In this study, F1 still had some of the root galls, which indicated that F1 exhibited a medium level of resistance to nematodes. A previous study [18] explained that resistant plants can reduce the symptoms caused by nematodes such as root gall induction, inhibit nematode survival, and stop them from making feeding sites.

Based on the peroxidase activity analysis in the present study, the resistant plants showed a higher value of peroxidase activity than the susceptible plants. A higher

concentration of peroxidase activity is required for plant resistance to protect themselves against the root-knot nematode. A plant that has the R (resistance) gene can protect itself from pathogen attack, and the protection is associated with the hypersensitive response (HR) such as cell death and production of reactive oxygen species (ROS) such as hydrogen peroxide (H_2_O_2_). H_2_O_2_ plays an important role in the inhibition of pathogens and in the formation of free radicals that are toxic to organisms including *M. incognita*. The formation of physical barriers such as lignification and suberization prevents the penetration of nematodes into the tissues [19]. Nematode juveniles that are surrounded by necrotic cells caused due to ROS production cannot survive and develop in the tomato root. They fail to penetrate and migrate in the cell because of plant resistance responses occurring early. H_2_O_2_ is toxic to both nematodes and plants, and resistant plants produce ROS-scavenging enzymes to neutralize the toxin [20].

The significant difference between the non-infected and infected treatment observed in this study indicates that peroxidase is increased in plant resistance after nematode inoculation. Souza et al. described that peroxidase activity increased in maize after inoculation with maize dwarf mosaic virus [21]. El-Argawy and Adss also described that tomato (var. Nicola) resistance increased drastically with peroxidase activity at 12 h after *Ralstonia solanacearum* inoculation [22]. Peroxidase contributes to the level of tomato plant resistance to the root-knot nematode by producing toxic metabolites, inhibiting nematode development and nematode penetration into the roots [10]. Purwar et al. also described that a high level of peroxidase activity correlates with the tomato resistance level [23].

Metabolomic analysis of the non-infected and infected treatment showed a separation between the resistant and susceptible plants. This finding indicated that there is a difference in the chemical compounds and the concentrations between the plants. Based on the loading plot analysis, the primary and secondary metabolites differentiated between the resistant and susceptible plants. These included α- and β-glucose (as the primary metabolites) and caffeic acid (as the secondary metabolite from the organic acid). Higher concentration of α- and β-glucose in the resistant genotypes indicated that sugars are important substrates in plant biochemical systems and act as signal molecules.

Ehness et al. explained that a glucose or sucrose treatment in *Chenopodium rubrum* caused defense response induction by increasing mRNA of phenylalanine ammonia-lyase [24]. They also reported that the application of sugar spraying in the soil medium affected the resistance potato plants against nematodes and decreased root galling. Another study showed that glucose is an important substrate required by the plant as a source of energy, plays a role in the formation and development of plant structures, and acts in the signaling pathways in plant defense mechanisms. It can also increase the localization of cell death around the infected area and the lignification of the cell wall [25]. Moghadam and Ede demonstrated the function of sugar compounds in the plant defense system, wherein they reported that it acts as a priming molecule generating pathogen-associated molecular patterns that trigger pathogen-associated molecular patterns-triggered immunity and effector-triggered immunity in plants [26].

Sugar compounds that resulted from the photosynthesis and carbon fixation process in plants play a core role in metabolic response to changes in the environment. Sugar sensing and signaling are complex processes in plant tissue that influence cell development and growth. There were studies that explained the synergy between jasmonic acid and glucose to modulate the accumulation of glucosinolate in *Arabidopsis*. Guo et al. explained that combined jasmonic acid and glucose regulate the accumulation of glucosinolates, whereas the glucosinolates are the group of secondary metabolites that have functions in antioxidant activity and resistance to biotic and abiotic stress [27].

This study also found that caffeic acid affected the classification between the susceptible and resistant genotypes. Caffeic acid is also one of the phenolic compounds that has been claimed to have a negative effect on nematode activity. Ohri and Satinder investigated ten phenolic compounds that were oxidized and assayed for nematicidal activity, of which oxidized α-resorcylic acid, ferulic acid, 3,4-dihydroxybenzoic acid and caffeic acid were found to have a high mortality effect on nematodes [28]. The amount of caffeid acid was higher in the resistant genotype (GM2) in the infected treatment, but there was no difference in caffeic acid between the genotypes in the control treatment. This suggests that only the most resistant genotype (GM2) showed an increasing trend in caffeic acid, wherein the concentration increased with an increase in nematode inoculation. F1 showed a lower caffeic acid concentration than the other genotypes in non-infected and infected treatment. This indicated that F1 did not have the same resistance mechanism as GM2 in terms of caffeic acid. Pegard et al. explained that caffeic acid, which is the product of hydrolytic enzymes such as esterases, is present in high levels in resistant tomato roots after nematode infestation and might contribute to the protection of plants against root-knot nematode infection [29]. Caffeic acid creates a toxic environment for nematode development and reproduction. Based on this finding, further study is required to reveal the effectiveness of caffeic acid against nematode development with *in vivo* bioassays.

The findings in this study were chemical compounds (peroxidase, α- and β-glucose, and caffeic acid) associated with tomato-nematode resistance mechanisms. Therefore, these compounds can be used as markers to select for resistance of tomato plants against nematodes. These compounds also can be used as induced resistance for tomato plants. Based on Ehness et al., the application of sugar spraying in the foliar plants can increase the level of resistance of potato plants and affect the development of nematodes in the root [24]. Therefore, the further studies are required to reveal the effectiveness of these compounds for resistance mechanisms.

## 5 Conclusion

Resistant and susceptible plants have different metabolite concentrations in response to infection by nematodes. The metabolites with a high concentration in the resistant plants were α- and β-glucose (sugar compounds from primary metabolites) and caffeic acid (aromatic region from secondary metabolites). Sugar plays a very important role in plant metabolism for the biosynthesis of essential compounds of cell and defense mechanism functions such as signaling pathways and enhancing resistance. Caffeic acid is a biological compound that helps the plant to inhibit the development of and increase the resistance against pathogens such as nematodes.
